# Expansion and safety profile of CD28‐costimulated anti‐CD19 CAR T cells in B‐cell lymphoma versus autoimmune disease

**DOI:** 10.1002/hem3.70426

**Published:** 2026-07-08

**Authors:** Mirjeta Berisha, Denise Walther, Martin Böttcher, Lea Reemts, Martin Mikuško, Enrico Schalk, Achim J. Kaasch, Romy Böttcher‐Loschinski, Ondrej Lukac, Tobias Hegelmaier, Stefanie Schreiber, Heiko Bruns, Stephan Fricke, Karolin Trautmann‐Grill, Verena Keitel, Aiden Haghikia, Dimitrios Mougiakakos

**Affiliations:** ^1^ Department of Hematology, Oncology, and Cell Therapy Otto von Guericke University Magdeburg Magdeburg Germany; ^2^ Healthcampus Immunology, Inflammation, and Infectiology (GC‐I3) Otto von Guericke University Magdeburg Magdeburg Germany; ^3^ Institute of Medical Microbiology and Hospital Hygiene Otto von Guericke University Magdeburg Magdeburg Germany; ^4^ Department of Neurology and Clinical Neurophysiology Hannover Medical School Hannover Germany; ^5^ Department of Neurology Otto von Guericke University Magdeburg Magdeburg Germany; ^6^ Magdeburg Center for Cell and Immune Therapy (MAZI) Otto von Guericke University Magdeburg Magdeburg Germany; ^7^ Department of Medicine 5, Hematology and Oncology Friedrich Alexander University Erlangen‐Nürnberg Erlangen Germany; ^8^ Institute for Clinical Immunology and Cell Therapeutics Otto von Guericke University Magdeburg Magdeburg Germany; ^9^ Fraunhofer Institute for Cell Therapy and Immunology Leipzig Germany; ^10^ Medical Clinic I, Hematology and Oncology TU Dresden Dresden Germany; ^11^ Department of Gastroenterology, Hepatology and Infectious Diseases Otto‐von‐Guericke University Magdeburg Magdeburg Germany

Autoimmune diseases (ADs) affect approximately 10% of the Western population, with an estimated 10%–15% of patients remaining refractory to standard‐of‐care therapies.^1^ In these individuals, disease manifestations are often associated with substantial impairment of quality of life and, in some cases, life‐threatening organ involvement, underscoring a clear unmet need for innovative therapeutic approaches.

Across a broad spectrum of ADs, B cells represent a central pathogenic driver. Beyond their role as producers of autoantibodies, B cells critically contribute to disease pathogenesis through antigen presentation, including the presentation of self‐peptides to autoreactive T cells, secretion of pro‐inflammatory cytokines, and dysfunctional regulation of immune tolerance, including defects in regulatory B‐cell subsets.^2^ Therapeutic strategies aimed at B‐cell depletion using monoclonal antibodies have therefore been extensively explored and have demonstrated clinical efficacy in selected indications, such as rheumatoid arthritis.^3^ However, these approaches have generally failed durable immune reset, limiting their transformative potential.

Cellular therapies targeting B cells, particularly chimeric antigen receptor (CAR) T cells, offer a fundamentally different strategy by inducing profound and sustained B‐cell depletion. In hematologic malignancies, anti‐CD19 CAR T cells are associated with deep and long‐lasting B‐cell aplasia,^4^ providing a strong biological rationale for their translation into ADs. Indeed, early case reports, case series, and more recently emerging clinical trial data have demonstrated striking clinical responses across multiple autoimmune indications, supporting the feasibility of this concept.^5^, ^6^, ^7^, ^8^ Similar signals have been reported in pediatric refractory AD, supporting broad applicability.^9^

Initial concerns that CAR T‐cell therapy might be associated with increased toxicity in autoimmune setting, given the presence of an inflammatory immune milieu, have so far not been substantiated. Instead, available evidence suggests a favorable tolerability profile in ADs, even superior to that observed in patients with hematologic malignancies.^10^ Hypothesized explanations include lower antigen burden, resulting in attenuated CAR T‐cell activation, although definitive mechanistic proof is currently lacking. Notably, in a recently published comparative analysis, patients with systemic lupus erythematosus (SLE) treated with second‐generation anti‐CD19 CAR T cells exhibited reduced toxicity compared with patients with lymphoma, who had received either CD28‐based or 4‐1BB‐costimulated CAR T cells, supporting the concept of disease‐context–dependent differences in tolerability.^11^

In this single‐center experience, we sought to extend these observations beyond SLE by focusing on patients with non‐lupus ADs treated under an extended clinical exception program. Six patients with treatment‐refractory ADs (three with myasthenia gravis, one with antibody‐associated encephalitis, one with immune thrombocytopenia, and one with IgG4‐related disease) received mivocabtagene autoleucel (miv‐cel), a second‐generation, CD28‐costimulated anti‐CD19 CAR T‐cell product with fully human binding domains, representing an optimized next‐generation derivative of axicabtagene ciloleucel (axi‐cel) (Figures 1A and S1).^12^ CAR T‐cell expansion kinetics and tolerability were compared with those of 20 consecutively treated patients with aggressive B‐cell non‐Hodgkin lymphomas (B‐NHLs) receiving axi‐cel. This design enabled a direct comparison of expansion and safety profiles across malignant and autoimmune indications using closely related CD28‐driven anti‐CD19 CAR T cells. Patient characteristics and methods are provided in the Supplemental Information. We first assessed *in vivo* expansion kinetics. Patients with ADs treated with miv‐cel exhibited more pronounced CAR T‐cell expansion compared with B‐NHL patients receiving axi‐cel. This was reflected by higher peak CAR T‐cell levels and an increased area under the curve. Peak expansion occurred slightly later in the AD cohort (Figure 1B–E). These differences cannot be explained by higher infused cell doses, as miv‐cel was administered at a lower, fixed target dose (1 × 10^8^ cells) compared to axi‐cel, which follows a weight‐based dosing strategy (2 × 10^6^ cells/kg body weight, capped at 2 × 10^8^ total cells). These findings are notable given the prevailing assumption that CAR T‐cell expansion is primarily driven by antigen availability. In ADs, where the overall CD19‐positive target cell burden is presumed to be lower than in aggressive B‐NHLs, attenuated expansion would have been anticipated. Instead, our data indicate that factors beyond absolute antigen load substantially influence CAR T‐cell expansion kinetics in this setting, potentially including intrinsic T‐cell fitness as well as construct‐related features. Notably, no differences in markers of mitochondrial dysfunction were observed between the CAR T cells in patients with ADs and B‐NHLs (Figure S2).^13^

**Figure 1:**
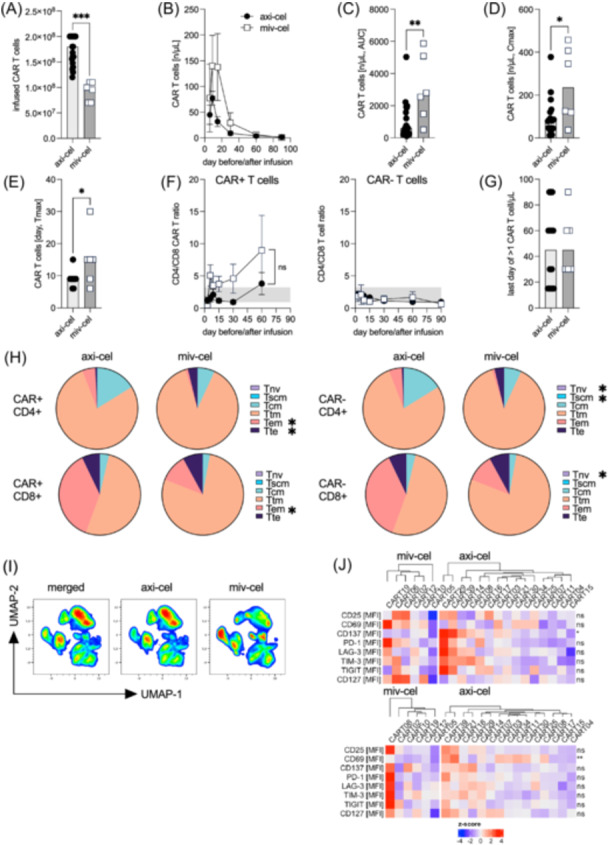
**Chimeric antigen receptor (CAR) T‐cell expansion kinetics and phenotypic characterization.** Comparison of patients with autoimmune diseases (ADs) treated with mivocabtagene autoleucel (miv‐cel, *n* = 6) and patients with aggressive B‐cell non‐Hodgkin lymphoma (B‐NHL) treated with axicabtagene ciloleucel (axi‐cel, *n* = 20). **(A)** Number of infused CAR T cells. **(B)** Longitudinal quantification of circulating CAR T cells following infusion. **(C)** Area under the curve (AUC) of CAR T‐cell expansion between the day of infusion (Day 0) and Day 90 after infusion. **(D)** Peak (=Cmax) CAR T‐cell levels. **(E)** Time to peak (=Tmax) CAR T‐cell expansion. **(F)** The CD4/CD8 ratio after infusion is shown for CAR‐positive T cells (left panel) and CAR‐negative T cells (right panel). The shaded gray area indicates the reference range of healthy donors. **(G)** Persistence is shown as the last time point at which ≥1 circulating CAR T cell per µL was detectable. **(H)** Pie charts show the distribution of T‐cell differentiation subsets in CAR‐positive (CAR^+^) and CAR‐negative (CAR^−^) CD4^+^ and CD8^+^ T cells at the time of peak CAR T‐cell expansion in axi‐cel‐ and miv‐cel‐treated patients. Differentiation subsets are defined as naïve (Tn), stem cell memory (Tscm), central memory (Tcm), transitional memory (Ttm), effector memory (Tem), terminal effector (Tte). **(I)** Uniform manifold approximation and projections (UMAP) of peripheral blood lymphoid cells (CD3^+^, CD4^+^, CD8^+^, CAR^+^, γδ TCR^+^, and CD56^+^) at the time of peak CAR T‐cell expansion are shown for the merged dataset (left), axi‐cel‐treated patients (middle), and miv‐cel‐treated patients (right), demonstrating comparable cellular distributions. **(J)** Heatmaps depict z‐score–normalized mean fluorescence intensity (MFI) of selected activation and inhibitory/exhaustion‐associated markers on CAR T cells at the time of peak expansion. Unsupervised hierarchical clustering was performed within the CAR T‐cell groups. Statistical significance is indicated as shown. Columns represent mean values; error bars indicate the standard error of the mean (SEM). Abbreviation: ns, not significant. *P ≤ 0.05, **P < 0.01, and ***P < 0.001.

Phenotypic subset analysis revealed a trend toward preferential expansion of CD4^+^ CAR T cells in the miv‐cel group, resulting in a shifted CD4:CD8 CAR T‐cell ratio compared with patients with B‐NHL. As CD4^+^ CAR T cells have been implicated in immune orchestration and treatment‐related toxicity, this observation is of potential relevance. Importantly, a comparable shift was not observed within non‐CAR‐expressing T cells, arguing against a global disease‐associated bias (Figure 1F). CAR T‐cell persistence did not differ significantly between groups, indicating that enhanced expansion in the autoimmune cohort did not translate into prolonged detectability (Figure 1G).

At the time point of peak CAR T‐cell expansion, phenotypic profiling revealed differences in differentiation status. Both CD4^+^ and CD8^+^ CAR T cells from axi‐cel‐treated patients exhibited a higher frequency of effector‐memory phenotypes compared with miv‐cel‐treated patients (Figure 1H). This pattern potentially reflects sustained antigen exposure in the context of higher target cell burden. The data for the miv‐cel group suggests that enhanced proliferation in this setting may not be directly coupled to accelerated effector differentiation. Analysis of activation and exhaustion markers demonstrated only modest differences between groups. Axi‐cel‐treated patients exhibited higher CD137 within CD4^+^ CAR T cells, as well as increased CD69 expression in CD8^+^ CAR T cells (Figures 1I,J and S3). These findings may indicate a slightly higher activation state in patients with lymphoma, although their biological significance remains uncertain given the overall similarity of activation profiles.

Taken together, these results suggest a partial dissociation between CAR T‐cell expansion kinetics and phenotypic activation or differentiation states, indicating that antigen burden alone may not fully account for disease‐context–dependent differences in CAR T‐cell behavior. These observations remain exploratory and warrant further investigation.

Cytokine release syndrome (CRS) occurred in the majority of patients in both cohorts, affecting 95% of patients with B‐NHL treated with axi‐cel and 63% of patients with ADs receiving miv‐cel. No CRS events exceeding grade 3 were observed in the miv‐cel cohort (Figure 2A). Consistent with this observation, the use of tocilizumab was more frequent in patients with B‐NHL, both in terms of the proportion of patients requiring intervention and the number of administered doses. This difference was paralleled by significantly higher serum interleukin‐6 concentrations measured on Day 6 after CAR T‐cell infusion (Figure 2B,C). Assessment of established parameters associated with CRS risk further supported a more pronounced inflammatory response in lymphoma patients.^14^ Baseline ferritin levels before initiating lymphodepletion were significantly higher in axi‐cel‐treated patients, and C‐reactive protein levels were at least numerically increased (Figure S4). In addition, we evaluated the modified Endothelial Activation and Stress Index (m‐EASIX), which incorporates lactate dehydrogenase, C‐reactive protein, and platelet counts.^15^ While no significant differences were observed, interpretation is limited by disease‐specific factors, particularly in patients with autoimmune cytopenias such as immune thrombocytopenia (Figure S5). With regard to immune effector cell‐associated neurotoxicity syndrome (ICANS), no events of any grade were observed in miv‐cel‐treated patients with ADs, whereas such events occurred in axi‐cel‐treated B‐NHL patients (Figure 2D). In line with the higher incidence of both CRS and ICANS, corticosteroids were also used more frequently and for a longer duration in the axi‐cel cohort (Figure 2E). One cardiopulmonary adverse event was observed in the axi‐cel cohort, whereas no such events occurred in miv‐cel‐treated patients.

**Figure 2:**
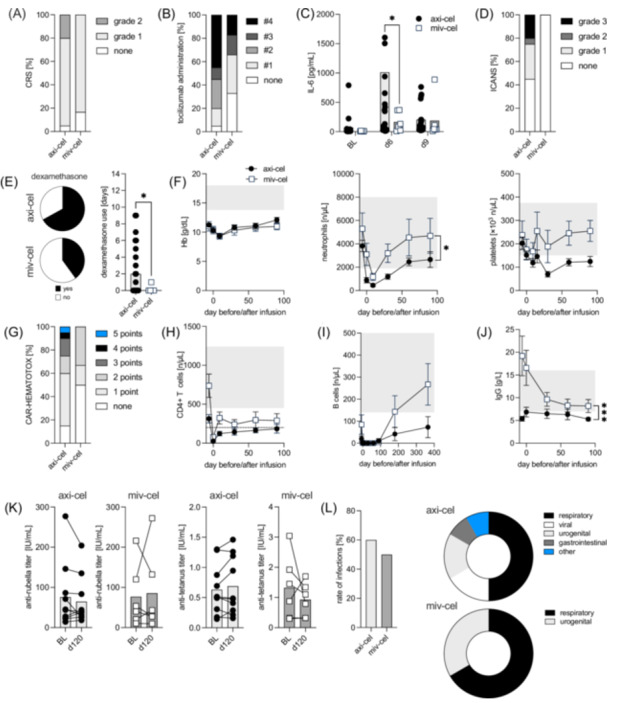
**Toxicity profile and hematopoietic and immune reconstitution following chimeric antigen receptor (CAR) T‐cell therapy.** Comparison of patients with autoimmune diseases (ADs) treated with mivocabtagene autoleucel (miv‐cel, *n* = 6) and patients with aggressive B‐cell non‐Hodgkin lymphoma (B‐NHL) treated with axicabtagene ciloleucel (axi‐cel, *n* = 20). **(A)** Incidence and maximum grade of cytokine release syndrome (CRS). **(B, C)** Serum interleukin‐6 (IL‐6) concentrations measured before initiating lymphodepletion (baseline, BL), on Day 6, and on Day 9 after CAR T‐cell infusion. **(D)** Incidence and severity of immune effector cell‐associated neurotoxicity syndrome (ICANS). **(E)** Proportion of patients receiving dexamethasone for CRS and/or ICANS (left panel) and cumulative duration of dexamethasone treatment (days) per patient (right chart). **(F)** Hematopoietic recovery over time, including hemoglobin levels, absolute neutrophil counts, and platelet counts. **(G)** CAR‐HEMATOTOX score prior to CAR T‐cell infusion. **(H)** Time to recovery of CD4^+^ T‐cell counts ≥200 cells/µL after infusion. **(I)** Longitudinal B‐cell reconstitution following CAR T‐cell therapy. **(J)** Total immunoglobulin G (IgG) levels over time after infusion. **(K)** Anti‐rubella and anti‐tetanus antibody titers measured at baseline (BL) and Day 120 after CAR T‐cell infusion in axi‐cel‐ and miv‐cel‐treated patients. **(L)** Incidence of infections in axi‐cel‐ and miv‐cel‐treated patients during follow‐up (left panel). Infection categories are indicated as shown in the right pie charts. Columns represent mean values; error bars indicate the standard error of the mean (SEM). Abbreviation: ns, not significant. *P ≤ 0.05, **P ≤ 0.01, and ***P ≤ 0.001.

Neutrophil and platelet reconstitution was more favorable in patients with AD, consistent with a greater bone marrow reserve and lower baseline inflammatory activity (Figure 2F). This was reflected by lower CAR hematotoxicity (CAR‐HEMATOTOX) scores in the miv‐cel‐treated cohort, likely attributable to disease biology and the absence of intensive cytotoxic pretreatment (Figure 2G).^16^ Accordingly, both the incidence and severity of early and late immune effector cell‐associated hematotoxicity (ICAHT) were reduced in autoimmune patients compared with axi‐cel‐treated lymphoma patients (Figure S6).^17^ In line with this observation, G‐CSF stimulation is less frequent in patients with AD (Figure S7).

Baseline lymphocyte counts prior to lymphodepletion showed a trend toward higher levels in patients with AD, potentially reflecting differences in prior treatments. Despite this, the overall kinetics of lymphocyte reconstitution appeared comparable between groups (Figure S8). Notably, miv‐cel‐treated patients reached and exceeded a CD4 T‐cell count of 200 cells/µL earlier, a threshold clinically relevant for the risk of opportunistic infections, viral reactivations, and the duration of anti‐infective prophylaxis (Figure 2H). No differences were observed in monocyte or NK cell reconstitution (Figure S9). Long‐term B‐cell reconstitution was superior in the AD cohort and was paralleled by higher immunoglobulin levels (Figure 2I,J). This interpretation is potentially tempered by elevated baseline immunoglobulins in AD patients, partly due to prior intravenous immunoglobulin administration and, in some cases, increased pathogenic autoantibodies (e.g., IgG4). Consistently, the need for immunoglobulin replacement was lower in the miv‐cel cohort compared to patients receiving axi‐cel, which was also reflected in fewer cumulative administrations (Figure S10). Despite these differences, no significant changes were observed in two exemplary protective antibody titers, rubella and tetanus (Figure 2K). This suggests that CD19‐based approaches, despite their impact on B cells and immunoglobulin levels, preserve protective antibody titers, which may in part be explained by the persistence of CD19‐negative long‐lived plasma cells.^18^ Impaired hematopoietic recovery, which is tightly linked to immune reconstitution, is associated with an increased risk of infections, a major contributor to non‐relapse mortality in CAR‐T‐cell–treated patients with malignant disease.^19^, ^20^ However, no differences in infection incidence were observed in our small cohorts (Figure 2L). Numerically, infectious complications in axi‐cel‐treated patients tended to occur later after infusion and included three cases of more severe infections requiring hospitalization, whereas no such events were observed in the miv‐cel group; however, these observations are based on very small numbers and should be interpreted with caution (Figure S11). No secondary malignancies were observed during follow‐up in either cohort, although follow‐up duration remains limited.

In summary, our data suggest disease‐context–dependent differences in CAR T‐cell expansion, toxicity, and immune reconstitution when comparing ADs and B‐NHLs using closely related CD28‐driven anti‐CD19 CAR T‐cell products. Our observations confirm and extend prior reports on CAR T‐cell tolerability in ADs beyond SLE.^11^ In line with the recently published analysis by Müller et al., patients with ADs in our cohort exhibited reduced inflammatory toxicity and more favorable hematopoietic recovery compared with patients with B‐cell lymphoma. However, whereas Müller et al. reported overall comparable CAR T‐cell expansion kinetics and shorter CAR T‐cell persistence in patients with SLE, we observed at least numerically enhanced expansion and comparable or potentially prolonged persistence in the AD cohort. These differences may reflect variations in the investigated autoimmune entities, as well as differences in CAR T‐cell constructs. In particular, miv‐cel incorporates fully human binding domains that were designed to reduce anti‐CAR immune responses and potentially support prolonged persistence.^12^ At the same time, the present analyses do not allow definitive separation of disease‐context effects from treatment‐ or product‐related factors. Similar to the observations by Müller et al., our data further suggest that differences in toxicity and immune reconstitution are unlikely to be solely explained by antigen burden, but may additionally be influenced by baseline inflammatory activity, bone marrow reserve, and prior treatment exposure, all of which differed substantially between the cohorts and represent important potential confounders. In addition, pharmacokinetic analyses of lymphodepleting agents and their active metabolites were not performed and may also influence CAR T‐cell expansion kinetics and toxicity.

Patients with ADs across rheumatologic, neurologic, and hematologic indications exhibited enhanced CAR T‐cell expansion, reduced inflammatory toxicity, and more favorable hematopoietic and immune recovery, without an increased risk of infections. However, given the limited patient numbers and the exploratory single‐center design, these findings should be interpreted with caution and require confirmation in larger multicenter cohorts. In addition, follow‐up remains limited with regard to long‐term complications such as secondary malignancies, and patient‐reported outcomes including quality‐of‐life assessments were not systematically collected. Future comparative studies should incorporate these clinically important endpoints. Collectively, these findings highlight the critical role of disease biology and treatment context in shaping CAR T‐cell behavior and support the continued development of CAR T‐cell therapy as a transformative strategy for refractory ADs.

## AUTHOR CONTRIBUTIONS

**Mirjeta Berisha**: Validation; writing—review and editing; formal analysis; data curation; methodology; investigation. **Denise Walther**: Methodology; validation; writing—review and editing; formal analysis; data curation; investigation. **Martin Böttcher**: Methodology; writing—review and editing; formal analysis; data curation; validation; investigation. **Lea Reemts**: Methodology; writing—review and editing; data curation; formal analysis; investigation. **Martin Mikuško**: Methodology; writing—review and editing; data curation; formal analysis; validation; investigation. **Enrico Schalk**: Methodology; writing—review and editing; formal analysis; data curation; validation; investigation. **Achim J. Kaasch**: Resources; data curation; formal analysis; writing—review and editing; methodology; investigation. **Romy Böttcher‐Loschinski**: Methodology; visualization; investigation; formal analysis; data curation. **Ondrej Lukac**: Investigation; methodology; formal analysis; data curation. **Tobias Hegelmaier**: Data curation; investigation. **Stefanie Schreiber**: Investigation; data curation. **Heiko Bruns**: Methodology; formal analysis. **Stephan Fricke**: Data curation; validation; writing—review and editing. **Karolin Trautmann‐Grill**: Investigation; data curation. **Verena Keitel**: Investigation; data curation. **Aiden Haghikia**: Investigation; data curation. **Dimitrios Mougiakakos**: Conceptualization; investigation; funding acquisition; writing—original draft; methodology; validation; visualization; writing—review and editing; formal analysis; project administration; data curation; supervision; resources.

## CONFLICT OF INTEREST STATEMENT

S.F. has received speaker honoraria, consulting fees, and travel support from Art tempi communications, BMS, Famicord, Johnson and Johnson, Kite/Gilead Sciences, MSGO, and Novartis Pharma. K.T.‐G. received speaker honoraria and consulting fees from Amgen, Grifols, Novartis, Roche, Sanofi, SOBI, Stemline, and Takeda, and travel support from NovoNordisk and SOBI. V.K. has received speaker honoraria and consulting fees from AstraZeneca, BMS, CSL, Falk, Gilead, GSK, Ipsen, and Mirum. A.H. has received speaker honoraria and consulting fees from AbbVie, Alexion, BMS, Galapagos, Johnson & Johnson, Kyverna Therapeutics, Merck Serono, and Sanofi. D.M. has received speaker honoraria and consulting fees from AbbVie, AstraZeneca, AvenCell, BMO Capital Markets, BMS, BeOne, Celgene, Galapagos, Gilead/Kite, Interius Bio, Janssen, Kyverna Therapeutics, Miltenyi Biotec, Novartis, and Roche, and travel support from AbbVie, AstraZeneca, BMS, BeOne, Janssen, Kyverna Therapeutics, Miltenyi Biotec, and Roche.

## FUNDING

H.B. and D.M. were supported by the Deutsche Forschungsgemeinschaft (536993790). D.M. was supported by the European Regional Development Fund (project ZELL‐THEMA). Open Access funding enabled and organized by Projekt DEAL.

## Supporting information

Supporting Information.

## Data Availability

Original data supporting the findings of this study are available from the corresponding author, Dimitrios Mougiakakos (dimitrios.mougiakakos@med.ovgu.de), upon reasonable request.

## References

[1] Conrad N , Misra S , Verbakel JY , et al. Incidence, prevalence, and co‐occurrence of autoimmune disorders over time and by age, sex, and socioeconomic status: a population‐based cohort study of 22 million individuals in the UK. Lancet. 2023;401(10391):1878‐1890. 10.1016/S0140-6736(23)00457-9 37156255

[2] Mougiakakos D , Meyer EH , Schett G . CAR T cells in autoimmunity: game changer or stepping stone? Blood. 2025;145(17):1841‐1849. 10.1182/blood.2024025413 39700499 PMC12782971

[3] Gürcan HM , Keskin DB , Stern JNH , Nitzberg MA , Shekhani H , Ahmed AR . A review of the current use of rituximab in autoimmune diseases. Int Immunopharmacol. 2009;9(1):10‐25. 10.1016/j.intimp.2008.10.004 19000786

[4] Brudno JN , Kochenderfer JN . Recent advances in CAR T‐cell toxicity: mechanisms, manifestations and management. Blood Rev. 2019;34:45‐55. 10.1016/j.blre.2018.11.002 30528964 PMC6628697

[5] Mougiakakos D , Krönke G , Völkl S , et al. CD19‐targeted CAR T cells in refractory systemic lupus erythematosus. N Engl J Med. 2021;385(6):567‐569. 10.1056/NEJMc2107725 34347960

[6] Haghikia A , Hegelmaier T , Wolleschak D , et al. Anti‐CD19 CAR T cells for refractory myasthenia gravis. Lancet Neurol. 2023;22(12):1104‐1105. 10.1016/S1474-4422(23)00375-7 37977704

[7] Müller F , Taubmann J , Bucci L , et al. CD19 CAR T‐cell therapy in autoimmune disease—a case series with follow‐up. N Engl J Med. 2024;390(8):687‐700. 10.1056/NEJMoa2308917 38381673

[8] Muller F , Hagen M , Wirsching A , et al. CD19 CAR‐T cells for treatment‐refractory autoimmune diseases: the phase 1/2 CASTLE basket trial. Nat Med. 2026;32(3):1142‐1151. 10.1038/s41591-025-04185-6 41501497 PMC13004673

[9] Becilli M , Metzler M , Bracaglia C , et al. Anti‐CD19 CAR T cells for pediatric patients with treatment‐refractory autoimmune diseases. Nat Med. 2026;32(3):1105‐1117. 10.1038/s41591-025-04191-8 41644747

[10] Schett G , Mackensen A , Mougiakakos D . CAR T‐cell therapy in autoimmune diseases. Lancet. 2023;402(10416):2034‐2044. 10.1016/S0140-6736(23)01126-1 37748491

[11] Müller F , Schwingen NR , Hagen M , et al. Comparison of the safety profiles of CD19‐targeting CAR T‐cell therapy in patients with SLE and B‐cell lymphoma. Blood. 2025;146(9):1088‐1095. 10.1182/blood.2025028375 40504989

[12] Brudno JN , Lam N , Vanasse D , et al. Safety and feasibility of anti‐CD19 CAR T cells with fully human binding domains in patients with B‐cell lymphoma. Nat Med. 2020;26(2):270‐280. 10.1038/s41591-019-0737-3 31959992 PMC7781235

[13] Rostamian H , Khakpoor‐Koosheh M , Fallah‐Mehrjardi K , Mirzaei HR , Brown CE . Mitochondria as playmakers of CAR T‐cell fate and longevity. Cancer Immunol Res. 2021;9(8):856‐861. 10.1158/2326-6066.CIR-21-0110 34344697

[14] Greenbaum U , Strati P , Saliba RM , et al. CRP and ferritin in addition to the EASIX score predict CAR‐T‐related toxicity. Blood Adv. 2021;5(14):2799‐2806. 10.1182/bloodadvances.2021004575 34264268 PMC8341350

[15] Pennisi M , Sanchez‐Escamilla M , Flynn JR , et al. Modified EASIX predicts severe cytokine release syndrome and neurotoxicity after chimeric antigen receptor T cells. Blood Adv. 2021;5(17):3397‐3406. 10.1182/bloodadvances.2020003885 34432870 PMC8525234

[16] Rejeski K , Perez A , Sesques P , et al. CAR‐HEMATOTOX: a model for CAR T‐cell‐related hematologic toxicity in relapsed/refractory large B‐cell lymphoma. Blood. 2021;138(24):2499‐2513. 10.1182/blood.2020010543 34166502 PMC8893508

[17] Rejeski K , Jain MD , Shah NN , Perales MA , Subklewe M . Immune effector cell‐associated haematotoxicity after CAR T‐cell therapy: from mechanism to management. Lancet Haematol. 2024;11(6):e459‐e470. 10.1016/S2352-3026(24)00077-2 38734026 PMC12413773

[18] Halliley JL , Tipton CM , Liesveld J , et al. Long‐lived plasma cells are contained within the CD19^−^CD38^hi^CD138^+^ subset in human bone marrow. Immunity. 2015;43(1):132‐145. 10.1016/j.immuni.2015.06.016 26187412 PMC4680845

[19] Rejeski K , Perez A , Iacoboni G , et al. The CAR‐HEMATOTOX risk‐stratifies patients for severe infections and disease progression after CD19 CAR‐T in R/R LBCL. J Immunother Cancer. 2022;10(5):e004475. 10.1136/jitc-2021-004475 35580927 PMC9114843

[20] Cordas Dos Santos DM , Tix T , Shouval R , et al. A systematic review and meta‐analysis of nonrelapse mortality after CAR T cell therapy. Nat Med. 2024;30(9):2667‐2678. 10.1038/s41591-024-03084-6 38977912 PMC11765209

