# Supplementary material for: Expansion and safety profile of CD28‐costimulated anti‐CD19 CAR T cells in B‐cell lymphoma versus autoimmune disease
Source: Hemasphere. 2026 Jul 8;10(7):e70426. doi: 10.1002/hem3.70426 (PMC13345674; doi:10.1002/hem3.70426)
Supplement: Supplementary file 1 — Supporting Information. [file HEM3-10-e70426-s001.pdf]

Supplementary Information

Materials and Methods

Patient samples

Peripheral blood from axi-cel/miv-cel-treated patients with underlying B cell-derived lymphoma (B-NHL) or autoimmune disorder (AD) was sampled longitudinally at predefined time points during the treatment course (day −6, day 0 [CAR T-cell infusion], and days +3, +6, +9, +15, +30, +60, and +90) after patient's informed consent (ethical vote 61-22 of the ethical committee at the Otto von Guericke University) and in accordance with the Declaration of Helsinki. Patient characteristics are detailed in **Supplemental Table 1**.

Peripheral blood mononuclear cells (PBMCs) were isolated by density-gradient centrifugation using Pancoll (PAN Biotech company, #P04-601000) and Leucosep™ tubes (Greiner bio-one, #227290), and biobanked in the vapor phase of liquid nitrogen until further use.

Toxicity grading and risk assessment

Cytokine release syndrome (CRS) and immune effector cell-associated neurotoxicity syndrome (ICANS) were graded according to the American Society for Transplantation and Cellular Therapy (ASTCT) consensus criteria[1]. Immune effector cell-associated hematotoxicity (ICAHT) was assessed according to established European Hematology Association (EHA)/European Society for Blood and Marrow Transplantation (EBMT) consensus criteria[2]. The modified Endothelial Activation and Stress Index (m-EASIX) and CAR-HEMATOTOX score were evaluated as previously described[3,4].

Multicolor flow cytometry

Diagnostic immunophenotyping and CAR T-cell monitoring were performed using the BD Multitest™ 6-color TBNK kit (BD Biosciences) and a panel of validated antibodies (see **Supplementary Table 2**), including the human CD19 CAR Detection Reagent (Miltenyi Biotec). Fresh peripheral whole blood samples were stained in BD Trucount™ Tubes according to the manufacturer's instructions.

For deep immunophenotyping, thawed PBMCs were stained with a viability dye (GhostDye® Violet 510, Cytek, or Zombie NIR™, BioLegend) and the human CD19 CAR Detection Reagent (Miltenyi Biotec). After Fc-blocking with Gamunex, cells were incubated with an antibody cocktail as detailed in **Supplementary Table 2**. For mitochondrial fitness assessment, cells were incubated with MitoTracker™ Green and MitoTracker™ DeepRed (both Thermo Fisher Scientific) for 30 minutes at 37°C and 5% CO_2 prior to surface marker staining. All reagents were used at pre-titrated concentrations according to the manufacturers' recommendations.

Data acquisition was performed on a Cytek Northern Lights 3L system following appropriate spectral unmixing using single-stained controls. Flow cytometry data were analyzed using FlowJo v10.10.0 (BD Biosciences, CA, USA).

For UMAP analysis, viable single cells were gated individually per patient, concatenated across patients, and batch-corrected using the CyCombine plugin. UMAP embeddings were generated using the UMAP_R plugin. $CD4^+$ and $CD8^+$ CAR^+ and CAR^- populations were defined based on CD4, CD8, and CD19-CAR expression (see **Supplementary Figure 3**) and used for downstream analyses. T-cell subsets were gated according to the strategy described by Mahnke et al[5].

LEGENDplex assay

Cytokine concentrations in patient serum were quantified using a customized bead-based multiplex immunoassay (LEGENDplex™, BioLegend, San Diego, CA, USA, #9000007684) according to the manufacturer's protocol. Undiluted patient serum samples were thawed on ice and directly used for analysis. Standard curves were generated using serial dilutions of the supplied standard cocktail. Data acquisition was performed on a Cytek Northern Lights 3L System and samples were analyzed using FlowJo V10.10. Analyte concentrations were calculated based on the generated standard curves.

Statistical Analysis

Normality was assessed using the Shapiro-Wilk test. As several datasets did not fulfill assumptions of normal distribution, comparisons between two groups were performed using two-sided Mann-Whitney tests. Mixed-effects models with the Geisser-Greenhouse correction were applied for longitudinal

analyses involving CAR T-cell product (axi-cel vs miv-cel) and time points, as appropriate. A p value ≤ 0.05 was considered statistically significant. All statistical analyses were performed using GraphPad Prism version 10 (GraphPad Software, San Diego, CA, USA).
Mean fluorescence intensities (MFI) of selected activation and inhibitory/exhaustion-associated markers on CAR T cells were imported into RStudio (R version 4.5.2) for statistical testing and heatmap generation using the ComplexHeatmap (version 2.24.1)[6,7] and rstatix (version 0.7.3)[8] packages.

References

1. Lee DW, Santomasso BD, Locke FL, et al. ASTCT Consensus Grading for Cytokine Release Syndrome and Neurologic Toxicity Associated with Immune Effector Cells. *Biol Blood Marrow Transplant*. Apr 2019;25(4):625-638. doi:10.1016/j.bbmt.2018.12.758
2. Rejeski K, Jain MD, Shah NN, Perales MA, Subklewe M. Immune effector cell-associated haematotoxicity after CAR T-cell therapy: from mechanism to management. *Lancet Haematol*. Jun 2024;11(6):e459-e470. doi:10.1016/S2352-3026(24)00077-2
3. Pennisi M, Sanchez-Escamilla M, Flynn JR, et al. Modified EASIX predicts severe cytokine release syndrome and neurotoxicity after chimeric antigen receptor T cells. *Blood Adv*. Sep 14 2021;5(17):3397-3406. doi:10.1182/bloodadvances.2020003885
4. Rejeski K, Perez A, Sesques P, et al. CAR-HEMATOTOX: a model for CAR T-cell-related hematologic toxicity in relapsed/refractory large B-cell lymphoma. *Blood*. Dec 16 2021;138(24):2499-2513. doi:10.1182/blood.2020010543
5. Mahnke YD, Brodie TM, Sallusto F, Roederer M, Lugli E. The who's who of T-cell differentiation: human memory T-cell subsets. *Eur J Immunol*. Nov 2013;43(11):2797-809. doi:10.1002/eji.201343751
6. Gu Z, Eils R, Schlesner M. Complex heatmaps reveal patterns and correlations in multidimensional genomic data. *Bioinformatics*. Sep 15 2016;32(18):2847-9. doi:10.1093/bioinformatics/btw313
7. Gu Z. Complex heatmap visualization. *Imeta*. Sep 2022;1(3):e43. doi:10.1002/imt2.43
8. Kassambara A. rstatix: Pipe-Friendly Framework for Basic Statistical Tests. 2025-10-18 doi:10.32614/CRAN.package.rstatix

Supplementary Figures

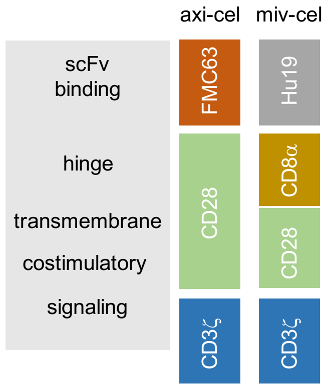

Supplemental Figure 1: Schematic representation of axi-cel and miv-cel CAR constructs. Both constructs comprise an anti-CD19 single-chain variable fragment (scFv), a hinge and transmembrane domain, a CD28 costimulatory domain, and a CD3ζ signaling domain. Axi-cel incorporates the murine-derived FMC63 scFv and a CD28 hinge/transmembrane region, whereas miv-cel utilizes a fully human anti-CD19 scFv (Hu19) with a CD8α hinge and a CD28 transmembrane region.

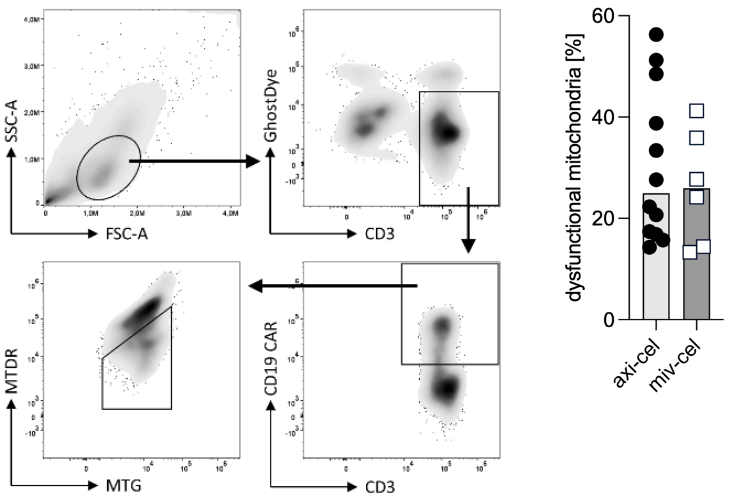

Supplemental Figure 2: Mitochondrial dysfunction in CAR T cells at the time of peak expansion in patients with aggressive B-cell non-Hodgkin lymphoma (B-NHL) treated with axicabtagene ciloleucel (axi-cel, n=20) and patients with autoimmune diseases (AD) treated with mivocabtagene autoleucel (miv-cel, n=6). The left panel shows a representative flow cytometry gating strategy selecting lymphocytes based on their FSC/SSC and viable T-cells gated on CD3^{+}GhostDye^{-} cells. Dysfunctional mitochondria in the CD19 CAR^{+} subset are defined by Mitotracker^{TM} Green (MTG) and MitoTracker^{TM} DeepRed (MTDR) as MTG^{high}/MTDR^{low}. The right panel depicts the frequency of dysfunctional mitochondria in circulating axi-cel and miv-cel CAR T cells at the time of peak expansion.
Columns represent mean values.

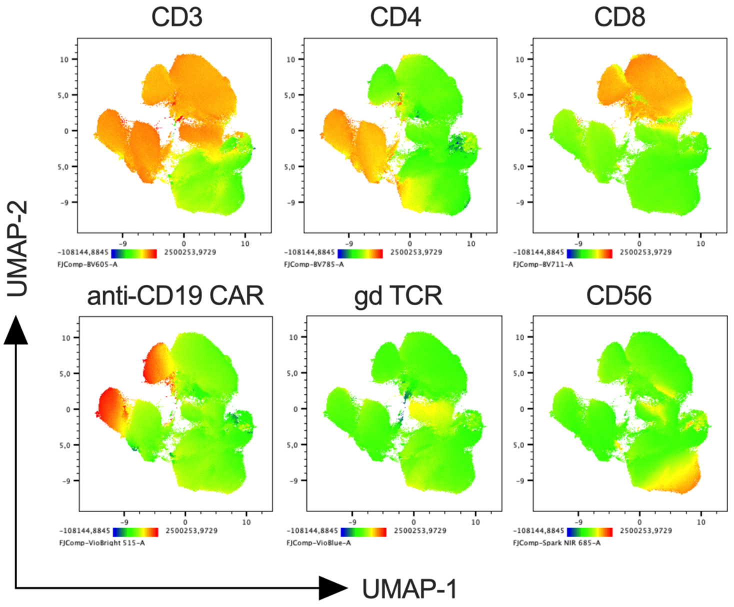

Supplemental Figure 3: Analysis of lymphoid cells at peak CAR T-cell expansion. Uniform manifold approximation and projections (UMAP) of peripheral blood lymphoid cells at the time of peak CAR T-cell expansion, colored by expression of CD3, CD4, CD8, anti-CD19 CAR, γδ TCR, and CD56.

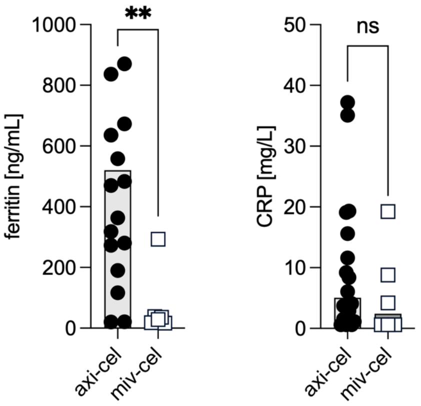

Supplemental Figure 4: Baseline inflammatory markers prior to lymphodepletion in patients with aggressive B-cell non-Hodgkin lymphoma (B-NHL) treated with axicabtagene ciloleucel (axi-cel, n=20) and patients with autoimmune diseases (AD) treated with mivocabtagene autoleucel (miv-cel, n=6). Columns represent mean values.

Abbreviations: ns, not significant; **, p≤0.01.

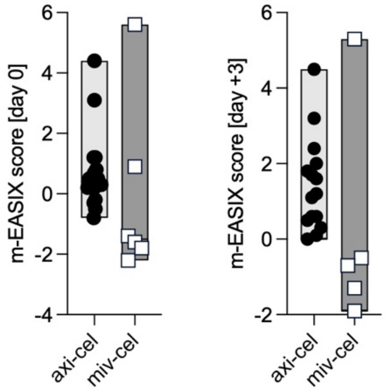

Supplemental Figure 5: Baseline toxicity-associated risk scores prior to CAR T-cell infusion in patients with aggressive B-cell non-Hodgkin lymphoma (B-NHL) treated with axicabtagene ciloleucel (axi-cel, n=20) and patients with autoimmune diseases (AD) treated with mivocabtagene autoleucel (miv-cel, n=6). Left panel: modified Endothelial Activation and Stress Index (m-EASIX) at day of CAR T-cell infusion (d 0). Right panel: m-EASIX at d +3.
Floating bars represent minimum-to-maximum values.

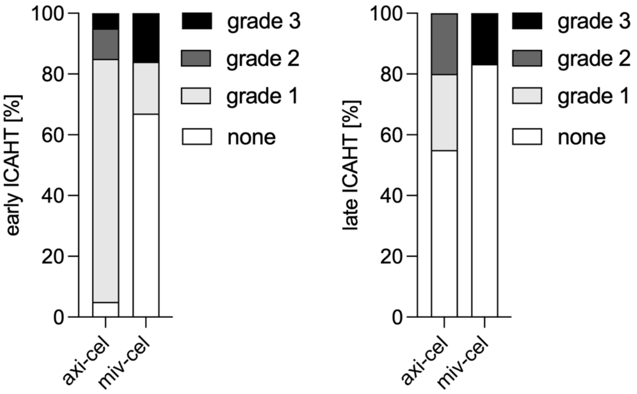

Supplemental Figure 6: Early and late immune effector cell-associated hematotoxicity (ICAHT) in patients with aggressive B-cell non-Hodgkin lymphoma (B-NHL) treated with axicabtagene ciloleucel (axi-cel, n=20) and patients with autoimmune diseases (AD) treated with mivocabtagene autoleucel (miv-cel, n=6). Stacked bar charts show the incidence and severity (grades 1-3) of early and late ICAHT in axi-cel- and miv-cel-treated patients.

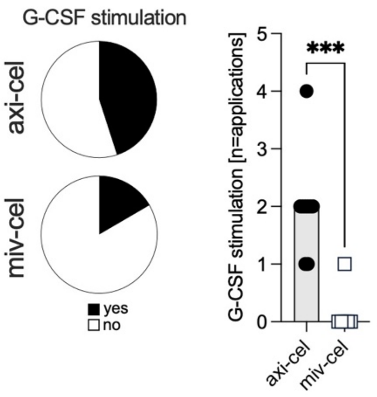

Supplemental Figure 7: Granulocyte colony-stimulating factor (G-CSF) use following CAR T-cell therapy in patients with aggressive B-cell non-Hodgkin lymphoma (B-NHL) treated with axicabtagene ciloleucel (axi-cel, n=20) and patients with autoimmune diseases (AD) treated with mivocabtagene autoleucel (miv-cel, n=6). Left panel: proportion of patients requiring G-CSF stimulation after CAR T-cell infusion. Right panel: cumulative number of G-CSF administrations among patients receiving G-CSF.
Columns represent mean values.
Abbreviations: ns, not significant; ***, p≤0.001.

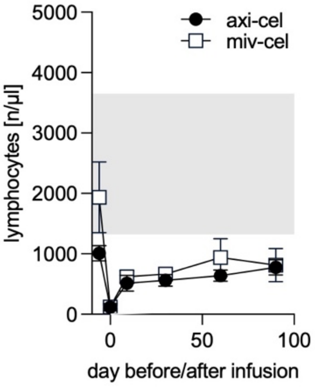

Supplemental Figure 8: Longitudinal lymphocyte reconstitution following CAR T-cell therapy in patients with aggressive B-cell non-Hodgkin lymphoma (B-NHL) treated with axicabtagene ciloleucel (axi-cel, n=20) and patients with autoimmune diseases (AD) treated with mivocabtagene autoleucel (miv-cel, n=6). Absolute lymphocyte counts were assessed before lymphodepletion and longitudinally after CAR T-cell infusion. The shaded gray area indicates the reference range of healthy donors. Error bars represent the standard error of the mean (SEM).

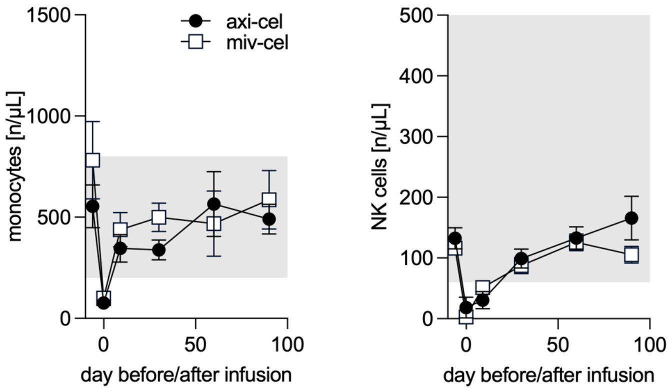

Supplemental Figure 9: Longitudinal reconstitution of circulating monocytes and natural killer (NK) cells following CAR T-cell therapy in patients with aggressive B-cell non-Hodgkin lymphoma (B-NHL) treated with axicabtagene ciloleucel (axi-cel, n=20) and patients with autoimmune diseases (AD) treated with mivocabtagene autoleucel (miv-cel, n=6). Absolute monocyte counts (left panel) and NK cell counts (right panel) in peripheral blood before and after infusion of axi-cel (open squares) or miv-cel (filled circles). Data are shown at indicated time points relative to CAR T-cell infusion. The grey shaded area indicates the reference range of healthy donors.

Error bars represent the standard error of the mean (SEM).

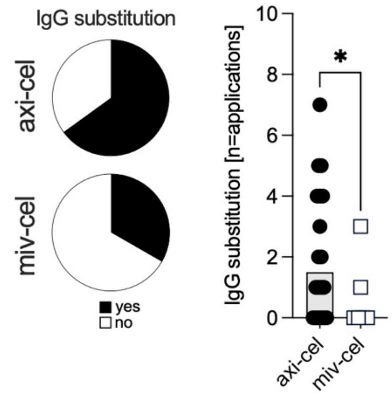

Supplemental Figure 10: Immunoglobulin G (IgG) replacement following CAR T-cell therapy in patients with aggressive B-cell non-Hodgkin lymphoma (B-NHL) treated with axicabtagene ciloleucel (axi-cel, n=20) and patients with autoimmune diseases (AD) treated with mivocabtagene autoleucel (miv-cel, n=6). Left panel: proportion of patients requiring immunoglobulin replacement therapy after CAR T-cell infusion. Right panel: cumulative number of immunoglobulin administrations among patients receiving replacement therapy.

Columns represent mean values.

Abbreviations: ns, not significant; *, $p \leq 0.05$.

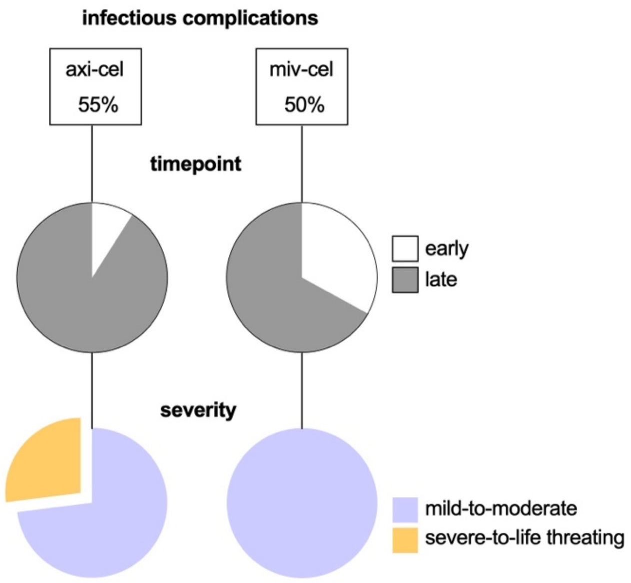

Supplemental Figure 11: Timing and severity of infectious complications following CAR T-cell therapy in patients with aggressive B-cell non-Hodgkin lymphoma (B-NHL) treated with axicabtagene ciloleucel (axi-cel, n=20) and patients with autoimmune diseases (AD) treated with mivocabtagene autoleucel (miv-cel, n=6). Upper panel: overall incidence of infectious complications during follow-up. Middle panel: timing of infections categorized as early (day 0 to +30 after CAR T-cell infusion) or late (>day +30). Lower panel: severity of infectious complications categorized as mild-to-moderate or severe-to-life threatening.

Supplementary Tables

Supplementary Table 1. Patients' characteristics

UPN	age	sex	diagnosis	number of previous therapies	lymphodepletion
CMD2024016	61	m	DLCBL	2	Flu + Cy
CMD2024015	54	m	DLBCL	2	Flu + Cy
CMD2024010	61	f	DLBCL	1	Flu + Cy
CMD2024011	45	m	MALT	2	Flu + Cy
CMD2024002	55	m	PCNSL	2	Flu + Cy
CMD2024001	52	f	DLBCL	2	Flu + Cy
CMD2024004	56	m	DLBCL	1	Flu + Cy
CMD2023014	47	m	FL	3	Flu + Cy
CMD2023020	63	m	DLBCL	1	Flu + Cy
CMD2023019	79	f	DLBCL	1	Flu + Cy
CMD2023018	64	m	DLBCL	2	Flu + Cy
CMD2023007	49	m	DLBCL	1	Flu + Cy
CMD2023005	49	m	DLBCL	2	Flu + Cy
CMD2024003	62	f	FL	2	Flu + Cy
CMD2023008	66	m	DLBCL	1	Flu + Cy
CMD2023009	60	m	PCNSL	1	Flu + Cy
CMD2023003	68	m	DLBCL	2	Flu + Cy
CMD2023002	43	m	DLBCL	2	Flu + Cy
CMD2025003	60	f	DLBCL	1	Flu + Cy
CMD2025013	53	m	DLBCL	1	Flu + Cy
CMD2023017	24	f	GMG	5	Flu + Cy
CMD2023012	36	f	GMG	5	Flu + Cy
CMD2023004	33	f	GMG	7	Flu + Cy
CMD2023011	59	m	IgG4-related disease	5	Flu + Cy
CMD2024007	35	m	ITP	7	Flu + Cy
CMD2023015	34	m	autoimmune encephalitis	3	Flu + Cy

Abbreviations: Cy, cyclophosphamide; DLBCL, diffuse large B-cell lymphoma; f, female; FL, follicular lymphoma; Flu, fludarabine; GMG, generalized myasthenia gravis; IgG4, Immunoglobulin G subclass 4; ITP, immune thrombocytopenia; m, male; MALT, extranodal marginal zone lymphoma of mucosa-associated lymphoid tissue; PCNSL, primary central nervous system lymphoma; UPN, unique patient number.

Supplementary Table 2. Antibody list for multiparametric flow cytometry.

Antigen	Fluorochrome	Clone	Isotype	Identifier	Distributor
**anti-human**					
CD45	FITC	HI30	Mouse IgG1, κ	555482	BD Pharmingen
CD3	BV605	OKT3	Mouse IgG2a, κ	317322	Biolegend
	APC-H7	SK7	Mouse IgG1, κ	641415	BD Biosciences
CD4	V500	SK3	Mouse IgG1, κ	647455	BD Horizon
	BV785	OKT4	Mouse IgG2b, κ	317442	Biolegend
CD8	V450	RPA-T8	Mouse IgG1, κ	560348	BD Horizon
	BV711	RPA-T8	Mouse IgG1, κ	301044	Biolegend
CD19 CAR-biotin	-	-	rec. Hum. IgG1	130-129-550	Miltenyi Biotec
Biotin	VioBright B515	REA746	rec. Hum. IgG1	130-110-957	Miltenyi Biotec
	PE	REA746	rec. Hum. IgG1	130-110-951	Miltenyi Biotec

CD45RO	PE	S19021B	Mouse IgG1, λ	376804	Biolegend
	APC	REA611	rec. Hum. IgG1	130-133-556	Miltenyi Biotec
CCR7	BV421	G043H7	Mouse IgG2a, κ	353208	Biolegend
CD95	BV510	DX2	Mouse IgG1, κ	305622	Biolegend
CD28	AF700	CD28.2	Mouse IgG1, κ	56-0289-41	Biolegend
TCRγδ	VioBlue	11F2	Mouse IgG1	130-113-507	Miltenyi Biotec
CD25	APC-Fire810	M-A251	Mouse IgG1, κ	356150	Biolegend
CD69	BV650	FN50	Mouse IgG1, κ	310934	Biolegend
CD137	PerCP-Fire806	4B4-1	Mouse IgG1, κ	309848	Biolegend
PD-1	PE-Fire640	EH12.2H7	Mouse IgG1, κ	329968	Biolegend
LAG-3	APC	REA351	rec. Hum. IgG1	130-119-567	Miltenyi Biotec
TIM-3	BV480	7D3	Mouse IgG1, κ	746771	BD Biosciences
TIGIT	PE-Cy7	A15153G	Mouse IgG2a, κ	372714	Biolegend
CD127	PerCP-Cy5.5	A019D5	Mouse IgG1, κ	351322	Biolegend
CD56	SparkNIR 685	5.1H11	Mouse IgG1, κ	362564	Biolegend
